# Microbiota Modulate Host Gene Expression via MicroRNAs

**DOI:** 10.1371/journal.pone.0019293

**Published:** 2011-04-29

**Authors:** Guillaume Dalmasso, Hang Thi Thu Nguyen, Yutao Yan, Hamed Laroui, Moiz A. Charania, Saravanan Ayyadurai, Shanthi V. Sitaraman, Didier Merlin

**Affiliations:** 1 Division of Digestive Diseases, Department of Medicine, Emory University School of Medicine, Atlanta, Georgia, United States of America; 2 Veterans Affairs Medical Center, Decatur, Georgia, United States of America; National Institute of Allergy and Infectious Diseases, National Institutes of Health, United States of America

## Abstract

Microbiota are known to modulate host gene expression, yet the underlying molecular mechanisms remain elusive. MicroRNAs (miRNAs) are importantly implicated in many cellular functions by post-transcriptionally regulating gene expression via binding to the 3′-untranslated regions (3′-UTRs) of the target mRNAs. However, a role for miRNAs in microbiota-host interactions remains unknown. Here we investigated if miRNAs are involved in microbiota-mediated regulation of host gene expression. Germ-free mice were colonized with the microbiota from pathogen-free mice. Comparative profiling of miRNA expression using miRNA arrays revealed one and eight miRNAs that were differently expressed in the ileum and the colon, respectively, of colonized mice relative to germ-free mice. A computational approach was then employed to predict genes that were potentially targeted by the dysregulated miRNAs during colonization. Overlapping the miRNA potential targets with the microbiota-induced dysregulated genes detected by a DNA microarray performed in parallel revealed several host genes that were regulated by miRNAs in response to colonization. Among them, Abcc3 was identified as a highly potential miRNA target during colonization. Using the murine macrophage RAW 264.7 cell line, we demonstrated that mmu-miR-665, which was dysregulated during colonization, down-regulated Abcc3 expression by directly targeting the Abcc3 3′-UTR. In conclusion, our study demonstrates that microbiota modulate host microRNA expression, which could in turn regulate host gene expression.

## Introduction

Host animals represent habitats for the diverse microbial ecosystems. The gastrointestinal tract, which harbors an abundant microbial population (10^14^ bacteria), is the most heavily colonized organ. Insights into the composition of microbial communities, microbe-host molecular interactions, and the impact of microbiota on developmental/functional features of the host have been acquired from studies on germ-free animals using genomic and associated computational methods [1], [2].

MicroRNAs (miRNAs), discovered in 1993 [3], [4], are small non-coding RNAs that post-transcriptionally regulate gene expression by binding to the 3′-untranslated regions (3′-UTRs) of target mRNAs [5]. Such binding is not homologous, allowing a single miRNA to potentially regulate hundreds of genes [6]. Increasing evidence has raised miRNAs as an important regulator of many cellular functions [5], [6], yet any role for miRNAs in microbiota-host interactions remains conjectural.

To address this topic, we investigated whether miRNAs are implicated in the gut microbiota-mediated regulation of host gene expression.

## Results and Discussion

### Microbiota modulate host miRNA expression

**Figure 1:**
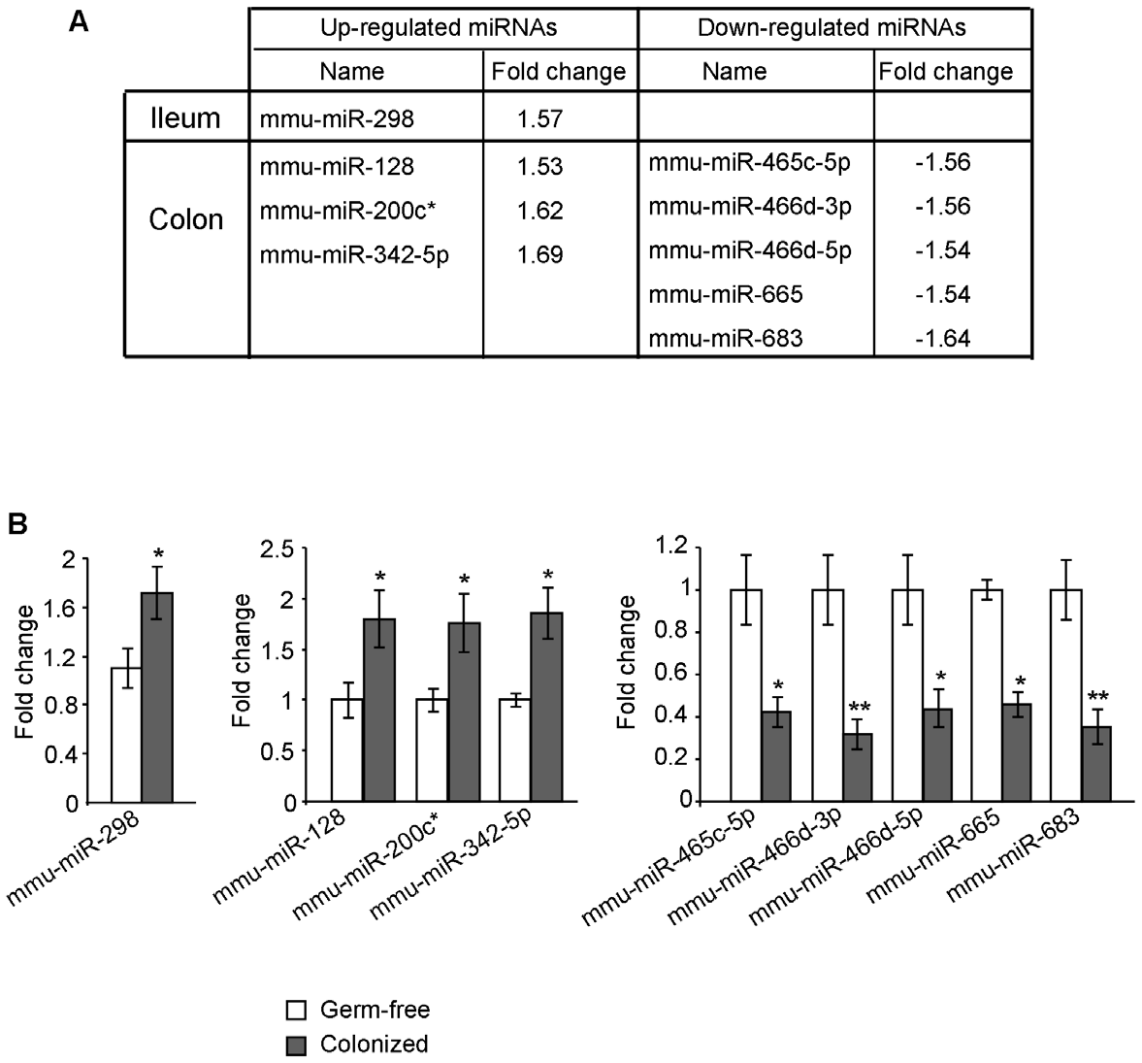
Microbiota modulate host miRNA expression. Germ-free mice were colonized with the microbiota from pathogen-free mice. Total RNAs were extracted from the ileums and colons of germ-free and colonized mice. MiRNAs differentially expressed in the ileum and colon of colonized mice compared to germ-free mice (≥ 1.5-fold) determined by miRNA array (A) and qRT-PCR (B). Values represent means ± S.E.M. (n = 6/group; *P<0.05; **P<0.005).

To determine if microbiota modulate expression of miRNAs in the host, germ-free mice were colonized with the microbiota from pathogen-free mice as previously described [7]. Comparative profiling of miRNA expression using miRNA arrays revealed significantly different signal intensities for 1 and 10 probe sets, representing one and eight miRNAs that were differently expressed in the ileum and the colon, respectively, of colonized mice compared to germ-free littermates (Figure 1A). Quantitative real-time RT-PCR (qRT-PCR) analysis confirmed the dysregulated expression of these miRNAs (Figure 1B).

Together, these data demonstrate that expression of host miRNAs is dysregulated in response to microbiota colonization.

### Computational identification of target genes of dysregulated miRNA during colonization

Genes potentially targeted by the colonization-induced dysregulated miRNAs were predicted using three algorithms miRanda, PicTar, and TargetScan (Tables S1, S2). Among eight miRNAs differentially expressed in the colon, seven were not in the PicTar database, and mmu-miR-200c* was not in the databases of any of the algorithms employed. To increase the stringency of the prediction, the Matchminer program was used to identify target genes that were predicted by at least two algorithms. This bioinformatic approach [8] revealed 164 potentially downregulated target genes for the upregulated mmu-miR-298 in the ileum (Table S3). Similarly, 342 genes potentially downregulated (targets of mmu-miR-128 and mmu-miR-342-5p) and 200 potentially upregulated genes (targets of mmu-miR-465c-5p, mmu-miR-466d-3p, mmu-miR-466d-5p, mmu-miR-665, mmu-miR-683) in the colon were identified (Table S4).

These potential miRNA target genes predicted by at least two algorithms will be compared with the genes that were dysregulated during colonization.

### MicroRNA-mediated regulation of host gene expression during colonization

To identify host genes dysregulated during microbial colonization, a DNA microarray was performed, exploring significantly different signal intensities for 124 and 302 probe sets, representing 97 and 241 dysregulated genes in the ileum (Table S5) and the colon (Table S6), respectively. It is worth to note that data obtained from miRNA arrays and DNA microarray revealed higher numbers of miRNAs and genes differentially expressed in the colon than that observed in the ileum, possibly reflecting bacterial load, which increases gradually from the stomach toward the small intestine to attain maximum in the colon [9].

**Figure 2:**
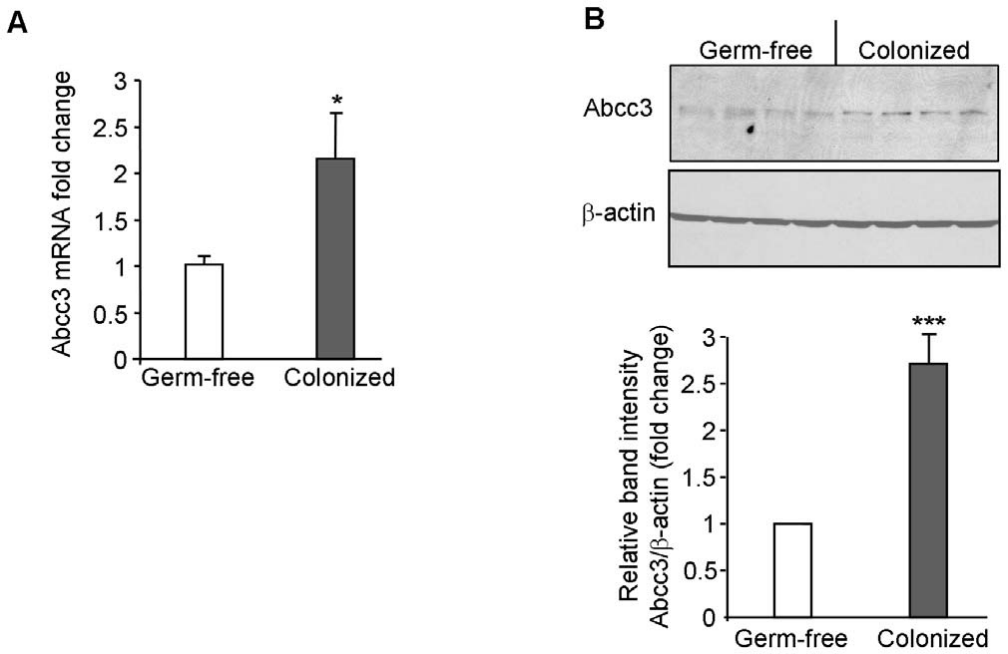
Microbiota up-regulate Abcc3 expression in mouse colon. Germ-free mice were colonized with microbiota from pathogen-free mice. (A) Total RNAs were extracted from the colons of germ-free and colonized mice. Abcc3 mRNA expression levels were assessed by qRT-PCR. Values represent means ± S.E.M. (n = 6/group; *P<0.05). (B) Abcc3 protein expression levels in the colons of germ-free and colonized mice were assessed by Western blot. Bar graphs in *B* show the relative intensity of blots (upper panel) with values represent means ± S.E.M. ***P<0.01.

Crossing the DNA microarray-detected dysregulated genes with the potential targets of dysregulated miRNAs predicted by at least two algorithms as described above revealed a single upregulated gene, Abcc3, potentially targeted by mmu-miR-665, for the colon, whereas no overlapping gene was found for the ileum. The up-regulation of Abcc3 during colonization was then validated by qRT-PCR (Figure 2A) and Western blotting (Figure 2B). Figure 2 shows that colonization of mice with microbiota significantly increased expression of Abcc3 in the colon at both mRNA and protein levels.

Together, by coupling a DNA array with a microRNA array accompanied with the computational approach, we identified Abcc3 as a dysregulated miRNA target gene during colonization.

### Mmu-miR-665 is directly involved in microbiota-induced Abcc3 up-regulation

**Figure 3:**
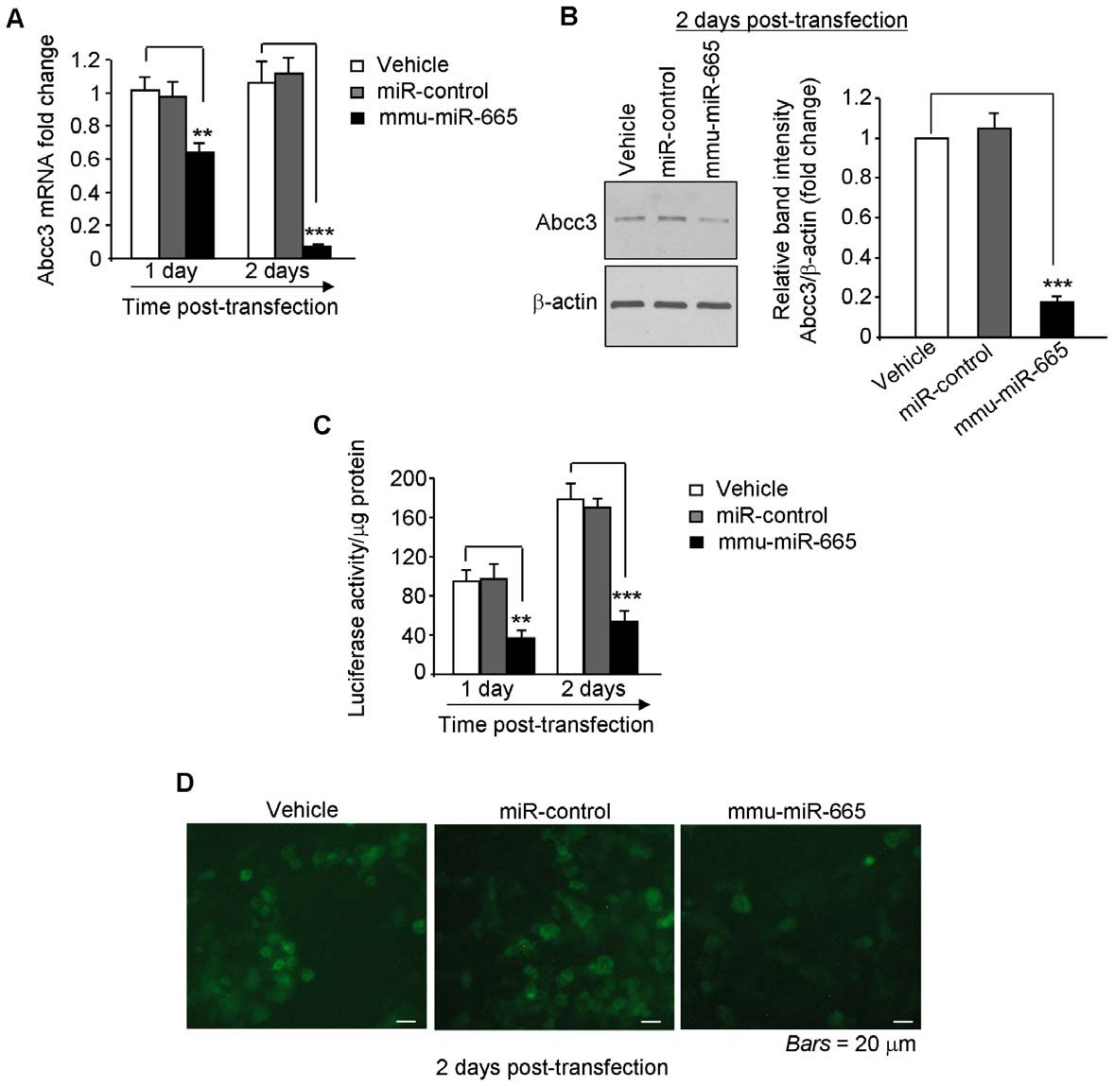
Mmu-miR-665 inhibits Abcc3 expression by directly targeting the Abcc3 mRNA 3′-UTR. (A, B) Mmu-miR-665 represses Abcc3 expression. RAW 264.7 cells were transfected with vehicle or precursors of miR-control or mmu-miR-665, and Abcc3 expression was assessed by qRT-PCR and Western blot. Bar graphs in *B* show the relative intensity of blots (left panel) from three independent determinations with values represent means ± S.E.M. (C, D) Mmu-miR-665 directly targets the Abcc3 mRNA 3′-UTR. RAW 264.7 cells were transfected with a luciferase or a GFP vector containing the Abcc3 3′-UTR in the presence or absence of mmu-miR-665. Luciferase activity (C) and fluorescent intensity (D) were determined. Values represent means ± S.E.M. (n = 6/group; *P<0.05; **P<0.005; ***P<0.001).

To directly examine the regulation of Abcc3 by mmu-miR-665, RAW 264.7 cells were transfected with vehicle, or a mmu-miR-665 precursor, or a control miRNA precursor. As shown in Figure 3A and B, mmu-miR-665 significantly inhibited Abcc3 expression at both mRNA and protein levels as assessed by qRT-PCR and Western blotting, respectively. In contrast, transfection of cells with vehicle or the control miRNA did not exhibit any significant effect on Abcc3 expression (Figure 3, A and B).

We then sought to investigate if mmu-miR-665 directly targets the 3′-UTR of Abcc3 mRNA. For this purpose, the Abcc3 3′-UTR was cloned downstream a luciferase- (Abcc3-luc) or a GFP-encoding sequence (Abcc3-GFP). RAW 264.7 cells were then transfected with Abcc3-luc or Abcc3-GFP constructs together with vehicle or mmu-miR-665 or miRNA-control. Mmu-miR-665 significantly repressed luciferase activity (Figure 3C) and GFP production (Figure 3D) in these cells, whereas vehicle or miR-control did not exhibit any significant effect. Collectively, these results demonstrate that mmu-miR-665 down-regulates Abcc3 expression by directly targeting the Abcc3 3′-UTR. Therefore, it is possible that microbiota upregulate Abcc3 expression by down-regulating mmu-miR-665. In other words, miRNAs might be involved in microbiota-regulated host genes expression.

It should be noted that our miRNA target prediction approach was extremely restrictive since i) only target genes predicted by at least two algorithms were crossed with the DNA microarray-detected dysregulated genes, and ii) some of the dysregulated miRNAs are absent in the databases of the algorithms employed. This markedly reduced the number of host genes potentially regulated by miRNA in response to microbiota. Nonetheless, the microbiota-dysregulated Abcc3 gene was identified as a potential miRNA target by two algorithms. Abcc3 belongs to the multidrug resistance-associated protein family, which mediates the metabolism of xenobiotics and endogenous toxins, an intestinal function dysregulated in response to colonization of mice [10]. In addition, it is worth to note that miRNAs can repress expression of proteins without affecting the mRNA levels [11], [12].

MiRNA target genes predicted by any of the three algorithms operated at lower stringency were compared with the DNA microarray-detected dysregulated genes, yielding higher numbers of overlapping genes (6 for the ileum and 50 for the colon) (Tables S7, S8). These genes might also represent potential microbiota-regulated miRNA targets and should be considered for future studies.

In summary, we demonstrate that microbiota modulate host miRNA expression. Thus, our study suggests an implication of miRNAs in microbiota-mediated host gene regulation.

## Materials and Methods

### Mice

8 week-old female Swiss Webster Germ-Free or Pathogen-Free were obtained from Taconic. Colonization was performed as previously described [7]. Pathogen-Free mice were introduced into Germ-Free mouse cage (ratio 1∶1) for 4 days and maintained in sterile cages. Mice were then sacrificed, and colonic and ileum samples were collected. All animal procedures were in accordance with Emory University Institutional Animal Care and Use Committee.

### Ethics statement

This study was carried out in strict accordance with the recommendations in the Guide for the Care and Use of Laboratory Animals of the National Institutes of Health. The protocol was approved by the Committee on the Ethics of Animal Experiments of the University of Emory (Permit Number: DAR-156-2008).

### Cell culture

RAW 264.7 cells were grown in Dulbecco's modified Eagle's medium media (DMEM, Invitrogen) supplemented with 14 mM NaHCO_3_, 10% fetal bovine serum, and 1.5 ìg/ml plasmocin (Invitrogen). Cells were kept at 37°C in a 5% CO_2_ atmosphere and 90% humidity.

### cDNA and miRNA expression analysis

Total RNAs were extracted from tissue samples using the RNeasy kit (Quiagen) according to the manufacturer's instructions. Concentration of the RNA samples were quantified by a Nanodrop spectrophotometer (NanoDrop Technologies, Inc), and RNA quality was assessed using the Agilent Bioanalyzer (Agilent Biotechnologies). The same RNA amounts from 2 mice were pooled into one sample. 3 samples/group (colonized mice or germ-free mice) were subjected to DNA microarray and miRNA array analysis.

DNA microarray was performed in triplicate using the Illumina MouseWG-6 Expression BeadChips, representing more than 45,200 transcripts. Briefly, RNA was amplified into cDNA and labeled by *in vitro* transcription using Illumina TotalPrep RNA Amplification Kit (Ambion, Applied Biosystems). The chips were processed as per manufacturer's instructions without any modification. The arrays were scanned using the BeadStation 500 Instrument (Illumina Inc.) and data were normalized using the GenomeStudio v1.0.2 (Illumina Inc.).

MiRNA array was performed in triplicate using the Illumina mouseMI_V2 chip, which contains up to 656 miRNAs, covering more than 96% of the miRNAs described in the miRBase database. MiRNA assay was performed as previously described according to the manufacturer's protocol [13]. Briefly, RNA was first polyadenylated using poly-A Polymerase (PAS, Illumina). The attached poly(A) tail was used for further priming of cDNA synthesis (CSS, Illumina) using a biotinylated oligo-dT primers contained a universal PCR primer sequence. Biotinylated cDNA was captured to a solid phase by streptavidin binding and further hybridized with miRNA-specific assay oligonucleotides. Assembled oligonucleotides were extended by DNA polymerase and subsequently eluted and added to a PCR reaction, in which one of the universal primers was fluorescently labeled and the other universal primer was biotinylated. The PCR products were captured on streptavidin beads, washed and denatured to yield single-stranded fluorescent molecules to hybridize the arrays. The arrays were scanned using the BeadStation 500 Instrument (Illumina Inc.) and data were normalized using the GenomeStudio v1.0.2 (Illumina Inc.).

### MiRNA target prediction

To determine the potential target genes of detected miRNAs, three different miRNA target prediction algorithms were used: PicTar (http://pictar.mdc-berlin.de) [14], miRanda (http://microrna.sanger.ac.uk/sequences/) [15] and TargetScan (http://www.targetscan.org/) [16]. The Matchminer program (http://discover.nci.nih.gov/matchminer/index.jsp) [17] was then used to determine genes that were identified by at least two algorithms as described previously [8].

### Quantitative real-time PCR (qRT-PCR)

Total RNAs isolated using the RNeasy kit as described above were reversely transcribed using the NCode^TM^ miRNA first-strand cDNA synthesis kit (Invitrogen) to quantify mature miRNA expression, or using the first-strand cDNA synthesis kit (Fermentas) to quantify Abcc3 expression according to the manufacturer's instruction. qRT-PCR was performed using SYBR Green qPCR Master Mix (Fermentas) on a Mastercycler Realplex^4^ (Eppendorf) using the following primers:

For mature miRNA expression: the universal primer provided in the NCode^TM^ miRNA first-strand cDNA synthesis kit was used together with one of the following forward primer:

mmu-miR-665: 5′-ACCAGG AGG CTG AGG TCC CT-3′

mmu-miR-128: 5′-TCACAGTGAACCGGTCTCTTT-3′

mmu-mir-200c*: 5′-CGTCTTACCCAGCAGTGTTTGG-3′

mmu-miR-342-5p: 5′-AGGGGTGCTATCTGTGATTGAG-3′

mmu-miR-466d-3p: 5′-TATACATACACGCACACATAG-3′

mmu-miR-466d-5p: 5′-TGTGTGTGCGTACATGTACATG-3′

mmu-miR-465c-5p: 5′-TATTTAGAATGGCGCTGATCTG-3′

mmu-miR-683: 5′-CCTGCTGTAAGCTGTGTCCTC-3′

mmu-miR-665: 5′-ACCAGGAGGCTGAGGTCCCT-3′

mmu-miR-298: 5′-GGCAGAGGAGGGCTGTTCTTCCC-3′

For Abcc3 expression:

Forward 5′-CTT CTT TTC CCG CTT GTC TTT-3′;

Reverse 5′- CCT CCT CAG ACA GAG ACC AGA-3′.

For expression of 36B4, used as a housekeeping gene:

Forward: 5′-TCCAGGCTTTGGGCATCA-3′;

Reverse: 5′-CTTTATCAGCTGCACATCACTCAGA-3′.

Fold-induction was calculated using the *C*t method: ΔΔCt  =  (*C*t_Target gene_-*C*t_housekeeping gene_)_group1_ - (*C*t_Target gene_-*C*t_housekeeping gene_)_group2_, and the final data were derived from 2^-ΔΔ*C*t^.

### Luciferase and GFP repression experiments

The 3′-UTR of Abcc3 mRNA was cloned into the *SpeI*/*HindIII* sites of the pMIR-REPORT^TM^ Luciferase vector (Ambion), or into the *XhoI*/*HindIII* of the pEGFP-C1 vector (BD Biosciences Clontech) using the following primers: Abcc3 *SpeI*: 5′-GCA CTA GTA TGA TGC ATT CCA AGG G-3′; Abcc3 *HindIII*: 5′-GCA AGC TTT GTT GAC TAC AGC TTT ATT TC-3′; Abcc3 *XhoI*: 5′-GCC TCG AGA TGA TGC ATT CCA AGG G-3′. For luciferase assay, RAW 264.7 cells cultured on 24-well plastic plates were co-transfected with 1 µg of the Abcc3 3′-UTR-luciferase construct and 40 nM of mmu-miR-665 precursor (PM 13124, Ambion) or pre-miR negative control (AM17110, Ambion) using lipofectamine 2000 (Invitrogen). Luciferase activity was measured at 1 or 2 days post-transfection using the Dual-Luciferase Reporter Assay system (Promega) and a Luminoskan Ascent luminometer (Thermo Electron Corp., Waltham, MA). Values were normalized to lysate protein concentration. For GFP repression experiment, RAW 264.7 cells seeded on coverslips were co-transfected with 1 µg of the Abcc3 3′-UTR-GFP construct and 40 nM of the indicated miRNAs using Lipofectamine 2000. After 2 days of transfection, GFP production was vizualized by fluorescent microscopy using a Zeiss Axioskop2 plus microscope.

### Western blot

Total proteins were extracted using RIPA buffer (150 mM NaCl, 0.5% sodium deoxycholate, 50 mM Tris-HCl [pH 8.0], 0.1% SDS, 0.1% Nonidet P-40, 2 mM Na_3_VO_4_, 10 mM NaF, supplemented with protease inhibitor cocktail [Roche]), resolved on polyacrylamide gels (Bio-Rad) and transferred to PVDF membranes (Bio-Rad). Membranes were blocked and then probed overnight at 4°C with anti-Abcc3 (Santa Cruz Biotechnology) or anti-β-actin antibody (Sigma). After several washes, membranes were incubated with the appropriate HRP-conjugated secondary antibodies. Immunoreactive bands were detected using the enhanced chimioluminescence detection system (Amersham Biosciences).

### Statistical analysis

Values were expressed as means ± S.E.M Statistical analysis was performed using unpaired two-tailed Student's *t*-test by InStat v3.06 (GraphPad) software. *P*<0.05 was considered statistically significant.

## Supporting Information

Table S1 Genes potentially targeted by mmu-miR-298, which is up-regulated in the ileum upon colonization, predicted by MiRanda, TargetScan or PicTar algorithms.
(XLS)

Table S2 Genes potentially targeted by miRNAs differently expressed in the colon upon colonization, predicted by MiRanda, TargetScan or PicTar algorithms.
(XLS)

Table S3 Target genes of mmu-miR-298 predicted by at least two of the three algorithms MiRanda, TargetScan and PicTar.
(XLS)

Table S4 Target genes of the miRNAs that were dysregulated in the colon upon colonization predicted by both miRanda and TargetScan algorithms.
(XLS)

Table S5 DNA microarray-detected genes that were differentially expressed in the ileum of colonized mice compared to germ-free mice.
(XLS)

Table S6 DNA microarray-detected genes that were differentially expressed in the colon of colonized mice compared to germ-free mice.
(XLS)

Table S7 DNA microarray-detected dysregulated genes that were potentially targeted by miRNAs differently expressed in the ileum upon colonization.
(XLS)

Table S8 DNA microarray-detected dysregulated genes that were potentially targeted by miRNAs differently expressed in the colon upon colonization.
(XLS)
